# Proteomic Analysis of Immature *Fraxinus mandshurica* Cotyledon Tissues during Somatic Embryogenesis: Effects of Explant Browning on Somatic Embryogenesis

**DOI:** 10.3390/ijms160613692

**Published:** 2015-06-15

**Authors:** Chun-Ping Liu, Ling Yang, Hai-Long Shen

**Affiliations:** State Key Laboratory of Tree Genetics and Breeding, School of Forestry, Northeast Forestry University, Hexing Road 26, Harbin 150040, China; E-Mails: liuchunping83@gmail.com (C.-P.L.); yangl-cf@nefu.edu.cn (L.Y.)

**Keywords:** manchurian ash, mass spectrometry, PCD, somatic embryos, stress response

## Abstract

Manchurian ash (*Fraxinus mandshurica* Rupr.) is a valuable hardwood species in Northeast China. In cultures of *F. mandshurica*, somatic embryos were produced mainly on browned explants. Therefore, we studied the mechanism of explant browning and its relationship with somatic embryogenesis (SE). We used explants derived from *F. mandshurica* immature zygotic embryo cotyledons as materials. Proteins were extracted from browned embryogenic explants, browned non-embryogenic explants, and non-brown explants, and then separated by 2-dimensional electrophoresis. Differentially and specifically expressed proteins were analyzed by mass spectrometry to identify proteins involved in the browning of explants and SE. Some stress response and defense proteins such as chitinases, peroxidases, aspartic proteinases, and an osmotin-like protein played important roles during SE of *F. mandshurica*. Our results indicated that explant browning might not be caused by the accumulation and oxidation of polyphenols only, but also by some stress-related processes, which were involved in programmed cell death (PCD), and then induced SE.

## 1. Introduction

Somatic embryogenesis (SE) is the process whereby somatic cells differentiate into embryos, which can then grow into regenerated plants. The development of somatic embryos closely resembles that of zygotic embryos as evidenced by their morphological characteristics at each developmental stage (globular, heart-shaped, torpedo-shaped, and cotyledon) [1,2]. Thus, SE is used as a model system to understand the mechanisms regulating plant embryogenesis [3]. In addition, because somatic embryos can be used for mass propagation of elite genotypes, and embryo quality and conversion frequency can be improved by optimizing culture conditions [4], SE is an ideal method for commercial cloning of genetically improved genotypes [5].

Molecular biology and proteomic techniques have provided new methods to study SE. Consequently, many genes and proteins related to SE have been identified [6,7,8]. These studies have increased our understanding of cell differentiation, development, and morphogenesis during SE. However, most of these studies focused on development of somatic embryos mainly, and fewer studies about “how to achieve somatic embryogenesis” [9,10].

Manchurian ash (*Fraxinus mandshurica* Rupr.) is a hardwood species in the genus *Fraxinus* in the Oleaceae, which is a valuable hardwood species in Northeast China. In our previous studies on SE in *F. mandshurica*, an interesting phenomenon was found: somatic embryos were produced mainly on browned explants. Our results showed that 67% of explants browned and over half of them (34%) produced somatic embryos, but only 3% of the non-brown explants (33%) produced somatic embryos. These results interested us because it is generally accepted that explant browning will result in a decline in culture competence, with eventual loss of totipotency [11] or even the death of explants. Explant browning is usually caused by oxidase; for example, polyphenol oxidase (PPO) oxidizes phenols to produce brown-colored ubiquinols, which accumulate in explants and are released into the medium. However, explant browning can also be caused by environmental stress or other adverse conditions, including programmed cell death (PCD) and natural death (necrosis). Pinto *et al.* [12] reported that phenol accumulation may play a role during the early stages of SE, but no further researches focused on this were reported. Our previous study indicated that SE of *F. mandshurica* was associated with the browning of explants, however, both the mechanism of browning and its relationship with SE are unknown.

We conducted a proteomics analysis using cultured explants of different characteristics, such as non-browned explants without somatic embryos (NBNSE), browned explants with somatic embryos (BSE) and browned explants without somatic embryos (BNSE), which were derived from *F. mandshurica* immature zygotic embryo cotyledons. The aims of this study is to clarify the important proteins that are associated with SE of *F. mandshurica*, and also, to understand why somatic embryos of the species are produced mainly on brown explants, or in other words, why the browned explants produce somatic embryos. We hope that our results may be useful for improving the SE system of *F. mandshurica* and will contribute to the theoretical basis of SE in woody plants.

## 2. Results and Discussion

### 2.1. Separation of Proteins

**Figure 1:**
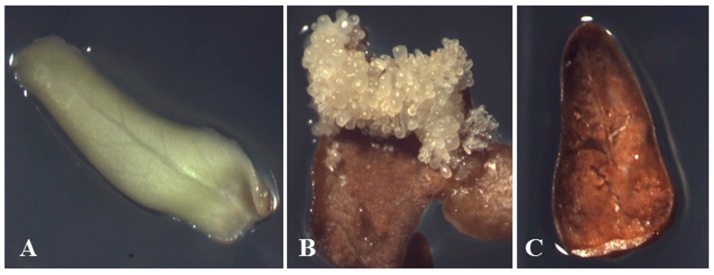
Explants derived from *Fraxinus mandshurica* immature zygotic embryo cotyledons with different characteristics after 40 days of culture. (**A**) non-browned explants without somatic embryos (NBNSE); (**B**) browned explants with somatic embryos (BSE) (somatic embryos were removed when sampling); and (**C**) browned explants without somatic embryos (BNSE).

**Figure 2:**
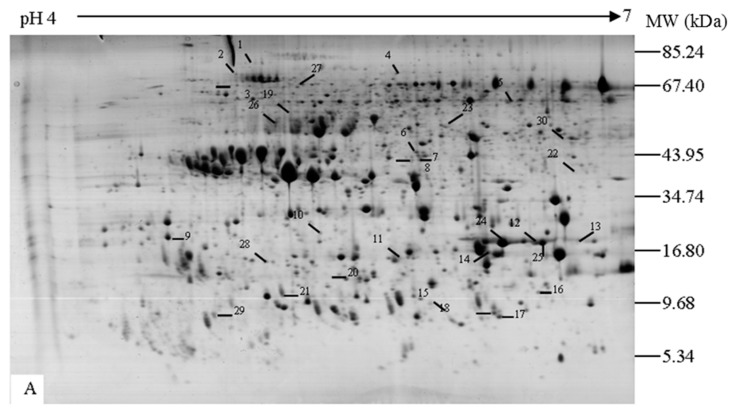

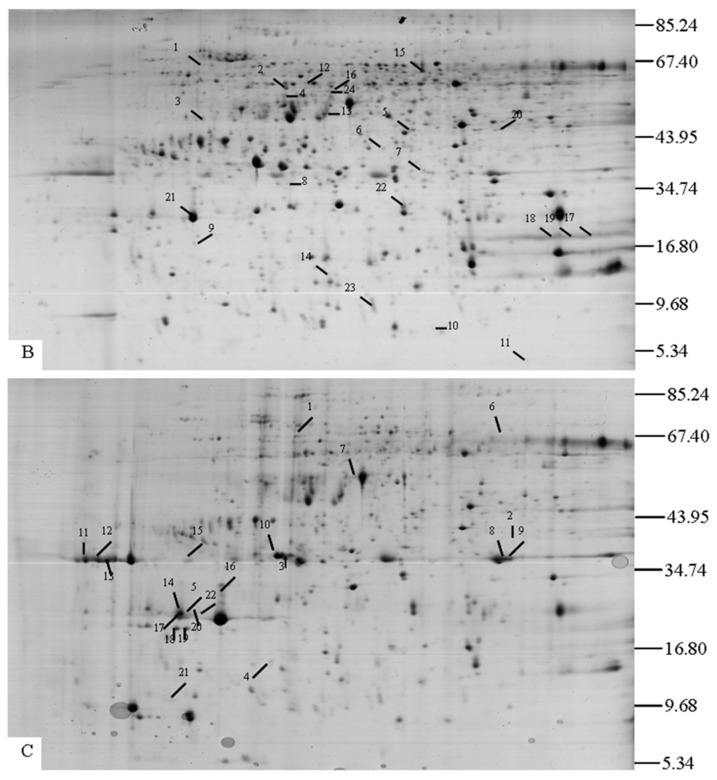
Protein spots expressed differentially and specifically from different explants of *Fraxinus mandshurica*. (**A**) Non-brown explant without SE, 1–23 show proteins specifically expressed in A, 24–30 show differentially expressed proteins between A and C (24 and 25 were up-regulated in A and 26–30 were down-regulated in A); (**B**) Browned explant with SE, 1–15 show specifically expressed proteins in B, 16–19 show differentially expressed proteins between B and C (16 and 19 were up-regulated in B and 17 and 18 were down-regulated in B), 20–24 show differentially expressed proteins between B and A (20–22 were up-regulated in B and 23–24 were down-regulated in B); (**C**) Browned explant without SE, 1–7, 10–16 and 20–21 show specially expressed proteins in C, 8 and 9, 17–19 and 22 show specially expressed proteins in browned explants (B and C), which were up-regulated in C.

**Figure 3:**
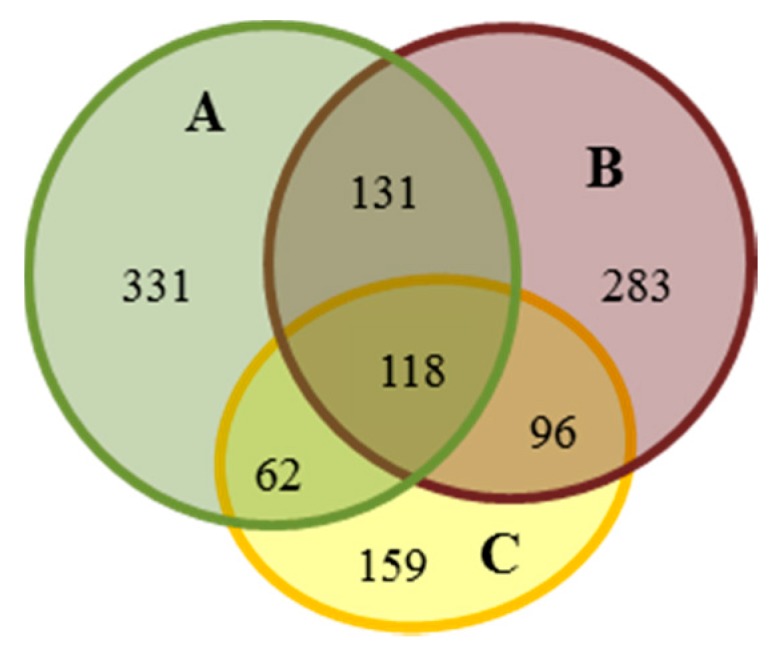
Venn diagram of protein spot assignment, which expressed in the three characteristics of explants that derived from *Fraxinus mandshurica* immature zygotic embryo cotyledons. (**A**) non-browned explants without somatic embryos (NBNSE); (**B**) browned explants with somatic embryos (BSE); and (**C**) browned explants without somatic embryos (BNSE).

The profiles of proteins extracted from NBNSE, BSE and BNSE explants (Figure 1) were evaluated by 2-DE. The number of reproducible protein spots in NBNSE, BSE, and BNSE was 642, 628, and 435, respectively (Figure 2 and Figure 3).

The proteins had pI values between 4 and 7 and *M*w values ranging from 5 to 100 kDa (Figure 2). Changes in protein abundance were analyzed by comparing %Vol values of spots among gels from different explant types. In total, 118 spots were common to all three experimental materials, of which up-regulated spots in B were 17 and eight compared to A and C, respectively; and up-regulated spots in C were 19 compared to A (Table 1). There were 180 spots common to NBNSE and BNSE, of which 49 were differentially expressed (35 up-regulated and 14 down-regulated in BNSE) (Figure 3, Table 1). The 49 spots were predicted to be involved in explant browning. There were 214 spots common to BSE and BNSE, 42 of which were differentially expressed: nine up-regulated and 33 down-regulated in BSE (Figure 3, Table 1). When the 214 spots in BSE and BNSE were compared with those in NBNSE, 96 protein spots were identified as being specifically expressed in the browned tissues (Figure 3). These were predicted to be involved in explant browning. Some of these protein spots expressed differentially and specially (Figure 2, Table 2) were identified by MALDI-TOF-TOF, and a database search was performed against NCBInr protein sequence databases with the combined MS + MS/MS ion searching program MASCOT (Available online: http://www.matrixscience.com).

**Table 1:** Protein spots expressed differentially and specially in non-browned explants without somatic embryos (NBNSE, A), browned explants with somatic embryos (BSE, B), and browned explants without somatic embryos (BNSE, C) during somatic embryogenesis of *Fraxinus mandshurica*.

Compared Gels	A				B				C			
	Specially	Differentially			Specially	Differentially			Specially	Differentially		
		up	mid	down		up	mid	down		up	mid	down
A-B	393	29	181	39	379	39	181	29	-			
B-C	-				414	9	172	33	221	33	172	9
A-C	462	14	131	35	-				255	35	131	14
ALL (A-B-C)	specially	differentially (A-B)			specially	differentially (B-C)			specially	differentially (A-C)		
	331	15	86	17	283	8	93	17	159	19	88	11

**Table 2:** Volume percentage (V%) of the protein spots expressed differentially in non-browned explants without somatic embryos (NBNSE, A), browned explants with somatic embryos (BSE, B), and browned explants without somatic embryos (BNSE, C) during somatic embryogenesis of *Fraxinus mandshurica*.

Spots	Differentially Expression (V%)		
	A	B	C
A24	0.735	-	0.088
A25	0.304	-	0.077
A26	0.062	-	0.240
A27	0.040	-	0.084
A28	0.065	-	0.254
A29	0.076	-	0.500
A30	0.025	-	0.103
B16	-	0.050	0.013
B17	-	0.024	0.110
B18	-	0.050	0.217
B19	-	0.108	0.031
B20	0.009	0.044	-
B21	0.171	1.148	-
B22	0.016	0.058	-
B23	0.133	0.019	-
B24	0.794	0.182	-
C8	-	0.168	0.305
C9	-	0.079	0.277
C17	-	0.119	0.333
C18	-	0.089	0.217
C19	-	0.055	0.223
C22	-	0.026	0.156

The number of spots such as A24 means the spot 24 in A in Figure 2, the same as below.

### 2.2. Functional Classifications of Identified Proteins

In total, 56 proteins representing 45 unique proteins were identified successfully. Proteins were assigned to eight functional categories by bioinformatic analyses: (1) carbohydrate and energy metabolism; (2) stress response; (3) protein metabolism/protein synthesis, assembly and degradation; (4) signal transduction; (5) nucleotide acid metabolism; (6) transcriptional regulation-related; (7) storage proteins; and (8) unknown function proteins (Table 3).

**Table 3:** Information about identified proteins derived from different explants during *Fraxinus mandshurica* somatic embryogenesis.

Spot ^a^		Matched Protein		gi Number ^b^	Organism	Mass/pI	Score ^c^	Queries Matched ^d^ (%)	Sequence Coverage ^e^ (%)
**Stress response and defense (18)**									
A2		Molecular chaperone Hsp90-1		38154489	*Lycopersicon esculentum*	80.1/4.98	100	31	35
A24		Dehydrin		18964	*Zea mays*	16.9/8.04	49	1	5
A25		Dehydrin		18964	*Zea mays*	16.9/8.04	64	1	5
A26		Peroxidase		1781326	*Spinacia oleracea*	38.2/6.15	212	3	7
A27		Heat shock protein, putative		223542544	*Ricinus communis*	71.1/6.10	684	17	23
B21		Osmotin-like protein		33340043	*Gossypium hirsutum*	26.6/7.41	63	1	4
C6		Peroxidase		1781326	*Spinacia oleracea*	38.2/6.15	177	3	20
C8		Chitinase precursor		5880843	*Petroselinum crispum*	29.0/6.3	94	5	30
C9		Chitinase precursor		5880843	*Petroselinum crispum*	31.3/4.88	117	6	32
C15		Chitinase precursor		5880843	*Petroselinum crispum*	29.0/6.3	109	5	30
C11		RecName: Full= Acidic endochitinase; Flags: Precursor		116332	*Solanum tuberosum*	31.3/4.88	63	5	10
C12		RecName: Full= Acidic endochitinase; Flags: Precursor		116332	*-*	29.0/6.3	68	9	38
C13		RecName: Full= Acidic endochitinase; Flags: Precursor		116332	*-*	31.3/4.88	64	4	10
C16		Putative thaumatin-like protein		53830843	*Solanum tuberosum*	25.0/5.31	79	2	6
C17		Putative thaumatin-like protein		53830843	*Solanum tuberosum*	25.0/5.31	62	3	12
C18		Putative thaumatin-like protein		53830847	*Solanum tuberosum*	27.2/8.32	149	6	22
C19		Osmotin-like protein		12274936	*Fagus sylvatica*	13.8/5.20	139	2	6
C22		Putative thaumatin-like protein		53830843	*Solanum tuberosum*	25.0/5.31	67	3	12
**Carbohydrate and energy metabolism (12)**									
A1		C4-specific pyruvate orthophosphate dikinase		31322756	*Miscanthus giganteus*	102.3/5.50	144	21	16
A8		GLX22(GLYOXALASE2-2); hydroxyacylglutahione hydrolase		15228389	*Arabidopsis thaliana*	28.8/5.93	66	3	18
A9		NADH dehydrogenase [ubiquinone]1 α subcomplex subunit 5 AltName: Full= NADH-ubiq		464258	*Solanum tuberosum*	4.1/9.41	72	4	30
A10		Lactoylglutathione lyase family protein, glyoxalase I family protein		42571377	*Arabidopsis thaliana*	15.4/6.2	71	1	40
A28	ATP synthase D chain, mitochondrial, putative			223530804	*Ricinus communis*	19.7/5.33	339	8	31
A30	GADPH (383 AA)			22240	*Zea mays*	40.9/7.21	950	18	39
B8	RecName: Full= triosephosphate isomerase, chloroplastic; short= TIM; short= triose-phosphate isomerase			1351271	*-*	34.4/6.45	245	23	37
B9	RecName: Full= ATP synthase subunit δ′, mitochondrial; AltName: Full= F-ATPasedelta’subunit; Fl			2493046	*-*	21.3/5.93	121	14	41
B11	Acyl-CoA-binding protein			19352190	*Panax ginseng*	9.90/5.43	118	4	18
B14	Putative NADH dehydrogenase (ubiquinone oxidoreductase)			21536893	*Arabidopsis thaliana*	56.7/8.87	64	17	37
B16	ATP synthase CF1 β subunit			11467199	*Zea mays*	54.0/5.31	730	13	34
C4	Mitochondrial F0 ATP synthase D chain			17939851	*Arabidopsis thaliana*	17.2/4.97	68	3	12
**protein metabolism (5)**									
A5	Aspartic proteinase			20800441	*Vigna unguiculata*	55.3/5.63	140	14	31
A6	Orf25			13449342	*Arabidopsis thaliana*	21.6/9.53	54	12	45
A7	Aspartic proteinase			20800441	*Vigna unguiculata*	55.3/5.63	140	4	4
A22	Aspartic proteinase			20800441	*Vigna unguiculata*	55.3/5.63	100	6	7
B7	Putative α7 proteasome subunit			14594925	*Nicotiana tabacum*	27.2/6.11	123	6	34
**Signal transduction (3)**									
A12	ANP3 (Arabidopsis NPK1-related protein kinase 3); kinase			15230612	*Arabidopsis thaliana*	71.6/8.41	48	14	24
A21	kinase associated protein phosphatase			3328364	*Oryza sativa*	63.3/6.66	64	15	23
B12	RPT3 (root phototropism 3); ATPase			15237159	*Arabidopsis thaliana*	45.7/5.42	124	12	24
**Nucleotide acid metabolism (2)**									
B4	DEAD box RNA helicase			25809054	*Pisum sativum*	46.9/5.39	270	16	33
B22	DEAD BOX RNA helicase RH15-like protein			8953379	*Arabidopsis thaliana*	49.3/5.43	273	6	16
**Transcriptional regulation (3)**									
A15			WRKY 14	34101231	*Theobroma cacao*	4.2/9.50	50	7	50
B15			Pentatricopeptide repeat (PPR)-containing protein	15228257	*Arabidopsis thaliana*	72.8/6.80	66	14	38
C10			tRNA-splicing endonuclease positive effector-related	15218807	*Arabidopsis thaliana*	120.2/8.82	58	3	12
**Storage proteins (2)**									
B19			7S globulin	13507023	*Elaeis guineensis*	66.3/6.53	74	1	2
B24			7S globulin	13507023	*Elaeis guineensis*	66.3/6.53	82	1	4
**Unknown function proteins (11)**									
A19			OSJNBb0034G 17.7	38605849	*Oryza sativa*	100.1/8.44	48	12	45
A13			Hypothetical protein	4678216	*Arabidopsis thaliana*	326.1/9.98	61	13	27
A23			Unknown protein	55168344	*Oryza sativa*	123.8/7.02	45	20	24
A29			Putative protein	4469015	*Arabidopsis thaliana*	72.1/4.87	56	1	1
B3			Os02g0698000	115448091	*Oryza sativa*	44.8/5.68	96	12	36
B5			Os07g0513000	115472339	*Oryza sativa*	39.7/8.6	64	17	37
B13			Unknown protein	42407715	*Oryza sativa*	26.3/11.63	104	8	22
B17			Unknown	194688752	*Zea mays*	47.2/5.95	361	8	20
B23			Hypothetical protein	147779485	*Vitis vinifera*	52.9/10.11	46	3	3
C2			Hypothetical protein	4090293	*Secale cereale*	19.5/8.8	64	13	54
C21			Os08g0500600	115477124	*Oryza sativa*	47.6/6.28	60	6	22

^a^ Spot numbers correspond to numbers indicated in Figure 2; ^b^ gi number in NCBI; ^c^ Probability based MOWSE score of MASCOT software for hit; ^d^ Number of matched peptide sequences identified via MASCOT; ^e^ Percentage of predicted protein sequence covered by matched peptides via MASCOT.

### 2.3. Proteins Playing Critical Roles in F. mandshurica Explant Browning and SE

#### 2.3.1. Stress Response and Defense Proteins

Of the 56 identified proteins, 18 were stress response and defense proteins. Somatic embryos are usually obtained from cultures under various stresses [13,14]. Although the relationship between stress and SE is not well understood, in some plant species, stress can reprogram somatic cells toward embryogenesis. For example, SE was induced using a high salt concentration, heavy metal ions, osmotic shock, and even wounding [13,15]. The roles of the identified stress response proteins and some other important proteins are discussed in detail below.

##### Chitinase

Chitinase, a glycosidase that catalyzes the hydrolysis of chitin, is a pathogenesis related (PR) protein that plays a major role in defense against different kinds of pathogens. Plants have evolved a range of defense mechanisms against pathogen infection, which could be mediated by resistance genes [16]. Once the specific recognition of pathogen avirulence gene and a corresponding resistance responses gene occurred, a response, which is known as the hypersensitive response (HR) was triggered at infection sites. Subsequently, the defense proteins, which are commonly referred as PR proteins, were induced [17]. Generally, chitinases expressed at low levels in certain organs at specific developmental stages [18], but was up-regulated by some stresses (e.g., SA (salicylic acid), H_2_O_2_, MeJA (methyl jasmonate) and ABA (abscisic acid) *etc.*) [19,20,21]. In our study, six spots (C8, C9, C11, C12, C13 and C15; Figure 2, Table 3) specifically expressed in browned explants (BNSE and BSE) were identified as chitinase precursors. The chitinase precursor expressed in browned explants but not in NBNSE, which indicated that it might be associated with explant browning. The browning of explants might be caused by some stress processes, which induced the chitinase finally. The process is a kind of HR, which involves the induction of programmed cell death (PCD), and results in the appearance of necrotic [17] and the browning of explants. Since all explants were cultured under the same condition, it is difficult to determine which stress the explants suffered from, except for a differential response of explants themselves to the stress, however, a further study is needed to clarify the issue.

Besides the defense activity, some chitinases also play roles in plant development, including the process of SE. De Jong *et al.* [22] identified an acidic chitinase that recovered the SE potential of a *ts11* temperature-sensitive mutant during carrot (*Daucus carota*) SE. Van Hengel *et al.* [23] suggested that endochitinase made arabinogalactan proteins (AGPs) more effective in promoting SE compared with non-chitinase-treated AGPs. Some chitinases were reported to express differentially during grapevine and chicory SE [24,25].

Although the mechanisms of how the PCD induced SE are not clear, two waves of programmed cell death occur during SE of Norway spruce have indicated that PCD played important roles in formation and development of somatic embryos [26]. In *F. mandshurica*, somatic embryos produced mainly on browned explants might be related to PCD. However, in our results, two (C8 and C9) of the six spots expressed in BSE were down-regulated in BSE compared to BNSE (Figure 2, Table 2), which seemed to indicate the decreased roles of the chitinase during later stage of SE of *F. mandshurica*.

##### Aspartic Proteinase

Three spots (A5, A7 and A22; Figure 2) specifically expressed in NBNSE were identified as aspartic proteinases. Ge *et al.* [27] reported that the aspartic protease encoded by the cell survival 1 (*PCS1*) gene in *Arabidopsis* may play a role as an anti-cell-death component by processing and activating a polypeptide that functions as a survival factor. The product of *PCS1* promoted cell survival during embryogenesis and gametogenesis, and ectopic over-expression of the *PCS1* gene blocked normal PCD processes associated with anther dehiscence. In contrast, the loss-of-function mutation of *PCS1* caused degeneration of both male and female gametophytes and resulted in excessive cell death in developing embryos. Thus, we can speculate that the expression of aspartic proteinase in NBNSE blocked PCD in explants and then inhibited explant browning. This implied that explant browning was caused by PCD. However, further research is required to confirm this assumption [27].

##### Dehydrins

Dehydrins are water-soluble lipid-associated proteins that accumulate during low-temperature or water-deficit, and play a role in freezing- and drought-tolerance in plants. In carrot, a dehydrin-like phosphoprotein, embryogenic cell phosphoprotein (ECPP-44), was present in embryogenic cells, stressed and non-stressed tissues, and somatic embryos, but was absent from non-embryogenic cells, indicating that ECPP-44 was expressed in specific tissues. ECPP-44 played a role in inducing and maintaining embryogenic cells [28]. In barley, similarly, *Dhn12*, which encodes an acidic YSK2 dehydrin, was specifically expressed in embryos, and not in response to stress [29]. In this study, two dehydrins (A24 and A25; Figure 2, Table 3) were differentially expressed between NBNSE and BNSE, and were down-regulated in BNSE. This result is interesting because dehydrins are stress-response proteins, and therefore, were expected to show up-regulated expression in stressed tissues. Similar result was reported in *Arabidopsis*, constitutive production of dehydrin ERD14 was also found to express in unstressed plants [30]. A probable explanation for these expressions is that constitutively expressed dehydrins could carry out some basic function/protection under unstressed growth conditions, or constitute a preformed defence required at the onset of stress [30]. Based on this, we speculated that the up-regulated expression of dehydrins in NBNSE played an important role to prevent the browning of explants.

##### Peroxidases

Two spots (spots A26 and C6; Figure 2) that were expressed differentially between NBNSE and BNSE or specifically in BNSE were identified as peroxidases. There were higher expression levels of these peroxidases in browned explants than in non-browned explants.

Peroxidases, a kind of PR proteins which belong to PR-9 family [16], were induced by wounding [31], pathogens [32], protoplast regeneration [33], oxidative stress [32,34] and plant development [35]. Takeda *et al.* [36] suggested that a peroxidase expressed during SE in asparagus (*Asparagus officinalis*) (AoPOX1) was secreted into cell walls where it catalyzed dimerization of coniferyl alcohol in the synthesis of dehydrodiconiferyl glucoside (DCG). DCG may play a role in activating cell division and differentiation during asparagus SE. Cordewener *et al.* [37] found that a cationic peroxidase overcame inhibition by tunicamycin, an inhibitor of glycosylation of secreted glycoproteins, which blocked SE before the globular stage during normal carrot SE. In longan [38] and cassava [39] SE, a peroxidase was found to express specifically. In addition, Richard-Forget and Gauillard [40] reported that peroxidases could enhanced degradation of polyphenol in pear when polyphenol oxidase existed, and phenol accumulation was reported to play a role during the early stages of SE in *Eucalyptus globulus*, although no further research focused on this were continued [12]. Thus, the higher expression levels in browned explants of peroxidases might be associated with the browning of explants and SE of *F. mandshurica* greatly.

##### Osmotin-Like Protein and Putative Thaumatin-Like Protein

Osmotin-like proteins and thaumatin-like proteins were also categorized as PR proteins, which belong to a family 5 of PR proteins [41]. Many PR5 proteins have been confirmed to possess antifungal activity in *in vitro* experiments, and some PR5 like proteins were also induced by various phytohormones, such as ABA, SA and jasmonic acid (JA), and environmental stress such as wounding, cold temperature and high salinity [41].

Osmotin-like proteins (B21 and C19; Figure 2, Table 3) were identified in all types of explants, but were expressed at higher levels in browned explants (BSE and BNSE) than in non-browned explants (NBNSE) (Table 2). Thaumatin-like proteins were specifically expressed in browned explants (BSE and BNSE) and were up-regulated in BNSE (C17-18 and C22, Table 2). These results were similar to that of chitinases, and could also be explained to be caused by some unknown stress or the differential response of explants themselves to the stress, which resulted in a HR that induced PCD and the browning of explants.

On the other hand, although osmotin-like protein has been detected during SE of chicory [25] and cassava [39], no reports to date have proposed a role for these proteins in SE.

##### Heat Shock Proteins (HSPs)

Heat-shock proteins (HSP) are absent or scarce in non-stressed conditions but are synthesized in response to various stresses, including temperature stress. Two heat shock proteins were expressed in BNSE and NBNSE. One was up-regulated in BNSE (spot A27) and the other was specifically expressed in NBNSE (spot A2). The HSP in spot A2 may function as a molecular chaperone. Györgyey *et al.* [42] considered HSPs as functional components in various assembly processes during embryogenic cell differentiation. In white spruce somatic embryos, two cDNAs encoded putative HSP-like proteins both showed developmental regulation under normal culture conditions. In addition, an HSP was detected during cassava SE [43]. It is believed that HSPs have specific roles in plant developmental pathways as well as in protection of embryos from heat-shock stress [44]. However, the functions of HSPs in plant embryogenesis have not been fully resolved.

#### 2.3.2. Signal Transduction

##### *Arabidopsis* NPK1-Related Protein Kinase 3 (ANP3)

The ANPs share high sequence similarity with tobacco NPK1, which is involved in cell cycle regulation [43]. Kovtun *et al.* [45] found that an ANP1 was induced specifically by H_2_O_2_ and activated a specific stress-induced MAPK. The activated MAPK cascade activates stress-response genes that protect plants from diverse environmental stresses, and also it represses auxin-inducible promoters. However, several H_2_O_2_-induced MAPK cascades regulate cell cycle progression in non-stressed cells [46], and oxidative stress can block cell cycle progression [47]. In our study, ANP3 was expressed only in NBNSE (A12) but not in browned explants. We could not explain this result, although it is possible that the MAPK cascade activated by ANP1 played a role in preventing browning.

##### Kinase-Associated Protein Phosphatase (KAPP)

The kinase-associated protein phosphatase (KAPP) interacts with receptor-like kinases (RLKs). This interaction is a key step in signal perception and transduction [48,49]. Both genetic and biochemical approaches have shown that KAPP negatively regulates RLK signaling [49,50]. A leucine-rich repeat (LRR)-RLK encoded by the somatic embryogenesis receptor kinase (*SERK*) gene was a marker for SE in a carrot suspension culture [2]. The *SERK* gene was expressed constantly during embryogenic cell formation in culture and during early embryogenesis [51]. In addition, Shah *et al.* [52] suggested that *At*SERK1 interacts with KAPP *in vitro*, and that KAPP dephosphorylated *At*SERK1 to prevent downstream components from receiving the *At*SERK1-mediated signal. This role of KAPP and the fact that KAPP was specifically expressed in NBNSE (A21) but not in browned explants may explain why the non-browned explants produced somatic embryos only rarely. The over-expression of *AtSERK1* mRNA resulted in three to four-fold increases in SE, which were sufficient to confer embryogenic competence. KAPP was expressed at greater levels in NBNSE than in BNSE. As described above, somatic embryos were mainly produced on browned explants; therefore, we predicted that the KAPP expression blocked the SERK-mediated signal and then restrained SE in *F. mandshurica*. The relationship between KAPP and SERK may provide a way to increase SE potential by suppressing KAPP activity.

#### 2.3.3. Other Proteins

Many other proteins involved in carbohydrate and energy metabolism, transcriptional regulation, and protein synthesis and degradation, and some proteins of unknown function were also expressed differentially or specifically in the different explants. There was no clear evidence for a relationship between these proteins and explant browning or somatic embryogenesis.

Some spots expressed specially in browned explants (BNSE and BSE, B9 and B16) or up-regulated in BNSE compared to NBNSE (A28) were identified as ATP synthase subunits. Yan *et al.* [53] reported that the expression of ATP synthase was up-regulated more than 100 times during apoptosis of hepatoma cells, indicating that ATP synthase may be involved in PCD.

In addition, a DEAD BOX RNA helicase RH15 and a similar protein were up-regulated in BSE compared with NBNSE. RNA helicases are highly conserved enzymes that modulate RNA structure. These enzymes participate in all biological processes involving RNA, including transcription, splicing and translation, and also embryogenesis, cell division, and differentiation [54,55]. Gong *et al.* [56] found that a mutant DEAD BOX RNA helicase gene was involved in plant development and the stress response in *Arabidopsis thaliana*.

Two protein spots (A8, A10) specifically expressed in NBNSE were identified as glyoxalase (GLO) family proteins. GLO is an intracellular enzyme found in the cytoplasm and organelles of prokaryotes and eukaryotes. In plants, the glyoxalase system consists of two enzymes: glyoxalase I (*S*-lactoylglutathionelyase), which catalyzes the conversion of methylglyoxal (MG) into its thioester in combination with coenzyme glutathione (GSH); and glyoxalase II (hydroxyacylglutathione-hydrolase) which catalyzes the hydrolysis of the thioester, regenerating GSH [57]. GLOs play a very important role in plant detoxification system. Ramaswamy *et al.* [58] found a correlation between glyoxalase-I activity and cell division/proliferation. Squaric acid and iso-ascorbate specifically inhibited the activity of glyoxalase-I [59]. Some studies have reported that MG is produced in excess in response to various stresses such as heavy metals and salinity stress in plants [60,61,62,63,64]. Yeast expressing a GLO gene derived from banana showed increased tolerance to abiotic stress [65].

## 3. Experimental Section

### 3.1. Materials Preparation

About 10,000 immature samaras from a ~50 year old tree of *F. mandshurica* were sampled in mid-July 2007 at Northeast Forestry University, Harbin, China. The samara wings were removed, after that the seeds were washed under running water for 2 h and then soaked in distilled water for 12 h. The seeds were disinfested in 2% NaClO (*v*/*v*) for 10 min after immersed in 70% ethanol for 30 s, and then washed five times with sterile water. Then, a segment of the seed coat (approximately 5-mm) was cut from the side of the hypocotyl using a sterile scalpel, and the immature cotyledon was removed using sterile forceps. Each single cotyledon was placed onto induction culture medium with its inside edge contacting the medium surface. The induction medium was one-half Murashige and Skoog (MS1/2) medium, in which all composition were half-strength of MS, containing sucrose (70 g·L^−1^), casein hydrolysate (CH, 400 mg·L^−1^), 6-benzyladenine (BA, 0.5 mg·L^−1^), and naphthaleneacetic acid (NAA, 1.5 mg·L^−1^). The pH was adjusted to 5.8 with 0.1 N NaOH, and 0.6% (*w*/*v*) agar was added before autoclaving at 121 °C for 20 min. The cultures were kept in a growth chamber at 24 ± 2 °C in darkness.

After 40 days of culture, we collected explants with the following characteristics: (1) non-browned explants without somatic embryos (NBNSE) (29.81% of total explants); (2) browned explants with somatic embryos (BSE) (34.06% of total explants, somatic embryos were removed when sampling); and (3) browned explants without somatic embryos (BNSE) (32.97% of total explants).

### 3.2. Protein Extraction

Protein extracts were prepared in biological triplicate for each explant. Proteins were extracted from approximately 200 mg tissue, which was ground to a fine powder in liquid nitrogen using a mortar and pestle. Then, the powder was added to a clear 10-mL tube containing 5 mL 12.5% (*w*/*v*) precooled TCA/acetone containing 20 mM DTT. The proteins were precipitated for 1 h at −20 °C, and then the mixture was centrifuged at 40,000× *g* for 15 min at 4 °C. The supernatant was discarded, and 5 mL cold acetone containing 20 mM DTT was added to the pellet. The mixture was kept for 2 h at −20 °C and then centrifuged at 40,000× *g* for 15 min at 4 °C. The addition of cold acetone and centrifugation steps were repeated 3 to 4 times and the supernatant was discarded. The precipitate was allowed to dry and then the powder was transferred into a 2-mL microtube. Then, lysis solution (7 M urea, 2 M thiourea, 40 mM DTT, 4% CHAPS, 2% pharmalyte 4–7) was added to the powder (15 μL lysis solution per mg powder), the mixture was shaken for 1 h, and then subjected to two 15-min supersonic treatments in an ice bath. Then, the mixture was centrifuged at 40,000× *g* for 15 min at 4 °C. Proteins in the supernatant were quantified by the Bradford assay (Bio-Rad, Hercules, CA, USA) with bovine serum albumin as the standard. The supernatants were stored at −80 °C or directly loaded for isoelectric focusing.

### 3.3. Two-Dimensional Gel Electrophoresis

Isoelectric focusing was performed using the IPGphor system (GE Healthcare, Milwaukee, WI, USA). We used 24-cm IPG strips with a broad-range pH 4–7 (GE Healthcare) for isoelectric focusing. Samples containing 1 mg proteins were used for 2-dimensional electrophoresis (2-DE). Rehydration buffer (7 M urea, 2 M thiourea, 2% CHAPS, 0.5% IPG buffer (pH 4–7), 0.002% bromophenol blue, and 2.8 mg·l-1 DTT freshly added before use) was added to the sample to complete the final volume to 450 μL. After 12 h of rehydration, isoelectric focusing was performed on an IPGphor II apparatus (GE Healthcare) using the following voltage-time program: 30 V for 8 h, 50 V for 4 h, 100 V for 1 h, 300 V for 1 h, 500 V for 1 h, 1000 V for 1 h, and 8000 V for 12 h. IPG strips were then subjected to reduction and alkylation by 2 × 20 min incubations with buffer (50 mM Tris-HCl (pH 8.8), 6 M urea, 30% glycerol, 2% SDS, 0.002% bromophenol blue) containing 1% DTT for the first incubation and 2.5% iodoacetamide for the second incubation. Sodium dodecyl sulfate-polyacrylamide gel electrophoresis (SDS-PAGE) was performed using 30.8% polyacrylamide gels (containing 0.8% bis-acrylamide) and an Ettan DALTsix electrophoresis unit 230 (GE Healthcare). Gels were stained with 0.04% (*w*/*v*) Coomassie blue R-350 (GE Healthcare) in 10% acetic acid and then destained with 10% acetic acid [66].

### 3.4. Spot Matching

Gels were scanned using a PowerLook 2100XL scanner (UMAX, Xinzhu, China) and analyzed using Image Master 2D Platinum software (version 5.0; GE Healthcare). The spot detection parameters were optimized by checking different protein spots in certain regions of the gel and then adjusting the automatic detection. Then, after visual inspection of the gels, we removed spots detected by mistake and added spots that were not detected by the software. To identify differentially and specifically expressed proteins, we first compared the spots among the different gels visually, and recorded spots present in all gels and calculated the %Vol (volume of a spot divided by the total volume in the whole gel, where the Vol is the integration of intensity of the spot over the spot area), pI, and *M*w. Then, we compared spots that showed differential expression (ratio of %Vol of the same spot was >2 or <0.5) between different explant types or specific expression in certain explant types. We compared all explant types with each other; that is, NBNSE to BSE, NBNSE to BNSE, and BSE to BNSE.

### 3.5. In-Gel Digestion of Protein Spots

Protein spots that were differentially and specifically expressed were excised from the gels and placed into 200 μL microtubes, then destained with 100 μL of a solution containing 25 mM ammonium bicarbonate and 50% acetonitrile for 30 min (twice), and then dehydrated in 50% acetonitrile for 15 min. The solution was discarded and the gel fragments were washed with 100% acetonitrile until they became white. The acetonitrile was discarded and the gel fragments were incubated in DTT solution (10 mM DTT in 25 mM ammonium bicarbonate) for 1 h at 56 °C. For alkylation, 50 μL 55 mM iodoacetamide in 25 mM ammonium bicarbonate was added and gel spots were incubated at room temperature in the dark for 45 min. After that, 50% acetonitrile was added to dehydrate the gel fragments and proteins were digested by 5 μL sequencing grade porcine trypsin (Promega, Madison, WI, USA) in an ice-bath for 30 min. The trypsin was discarded and 5 μL of 25 mM ammonium bicarbonate was added. The mixture was incubated overnight at 37 °C. Then, 2 μL 2.5% trifluoroacetic acid (TFA) was added and the samples were extracted for 1 h at 40 °C, and then re-extracted using 30 μL 5% TFA/50% acetonitrile for 1 h at 40 °C. The two extracted solutions were combined and evaporated using a Speed Vac, and then the residue was used for MS analysis. The MS/MS analyses were conducted by the National Center of Biomedical Analysis (Available online: www.peoteomics.cn).

### 3.6. Identification of Protein Spots by MALDI-TOF-TOF

The residue was purified and concentrated using ZipTip^®^ C18 (Millipore, Billerica, MA, USA), then was eluted by 1 μL solution containing 50% acetonitrile and 0.1% TFA. The elution was then loaded onto a target plate with 1 μL CHCA (5 mg/mL cyano-4-hydroxycinnamic acid), dried at room temperature. Mass spectral analysis was carried out using ABI 4800 MALDI-TOF-TOF MS (Applied Biosystems, Foster City, CA, USA). Mass ranges for MS peak were 800 to 4000 Da, and peaks were detected with the minimum signal/noise ratio of 20. MS survey scans were acquired in the positive-ion mode and accumulated at a laser intensity of 4000 with a wavelength of 355 nm and an accelerated voltage of 20 kV. Then, 10 peaks with the strongest signals were selected to obtain MS/MS data, with a laser intensity of 4100, an acceleration of 1 kV and a pressure of 3.2 × 10^−8^ Torr.

### 3.7. Mascot Database Search

A database search was performed against NCBInr protein sequence databases with the combined MS + MS/MS ion searching program MASCOT (Available online: http://www.matrixscience.com). The search parameters were set as follows, taxonomy: Viridiplantae; database: NCBInr; enzyme: trypsin; max missed cleavages: 1; fixed modifications: carbamidomethylation (C); variable modifications: oxidation (M), phosphorylation (STY); precursor tolerance: ±0.3 Da; MS/MS fragment tolerance: ±0.3 Da; mass type: monoisotopic; and peptide charge: H^+^.

## 4. Conclusions

SE is usually considered as an adaptive process of plant cells to stress [13]. In this study, most of the identified proteins expressed differentially and specifically in the three explants, especially the browned explants, were stress-response or defense proteins such as chitinases, peroxidases, aspartic proteinases, and an osmotin-like protein. No brown compounds were secreted into the media at the early stage of somatic embryo production. According to our previous study, the polyphenol content in *F. mandshurica* (1%) was not higher than that in *Syringa reticulate* var. *mandshurica* (0.7%) significantly*,* but the browning percentage of *F. mandshurica* was significantly higher than that of *S. reticulata* var. *mandshurica*, which indicated that the explant browning of *F. mandshurica* might be not related to accumulation of polyphenols greatly. Combined with the fact that the stress-response or defense proteins, especially the PR proteins, were specifically expressed in the browned explants, we predicted that the explant browning might be a manifestation of necrosis caused by some stress or by the differential response of explants themselves to the stress, which resulted in a HR that induced PCD and the browning of explants. Although the mechanisms for the PDC induction of SE are not clear, two waves of programmed cell death occur during SE of Norway spruce, which indicates that PCD played important roles in formation and development of somatic embryos [26]. This might be an explanation for the higher SE potential of browned explants than non-browned explants. Our results have highlighted an important issue about explant browning in plant tissue culture. Usually, browning is attributed to polyphenol accumulation, and therefore, many studies have concentrated on suppression of polyphenol accumulation and oxidation. Our results suggest that explant browning plays important roles during SE, at least in *F. mandshurica*, and therefore, it should not be prevented. Such results have not been reported before.
